# Prospect of Bioflavonoid Fisetin as a Quadruplex DNA Ligand: A Biophysical Approach

**DOI:** 10.1371/journal.pone.0065383

**Published:** 2013-06-13

**Authors:** Bidisha Sengupta, Biswapathik Pahari, Laura Blackmon, Pradeep K. Sengupta

**Affiliations:** 1 Department of Chemistry, Tougaloo College, Tougaloo, Mississippi, United States of America; 2 Biophysics Division, Saha Institute of Nuclear Physics, 1/AF Bidhannagar, Kolkata, West Bengal, India; Russian Academy of Sciences, Institute for Biological Instrumentation, Russian Federation

## Abstract

Quadruplex (G_4_) forming sequences in telomeric DNA and c-*myc* promoter regions of human DNA are associated with tumorogenesis. Ligands that can facilitate or stabilize the formation and increase the stabilization of G_4_ can prevent tumor cell proliferation and have been regarded as potential anti-cancer drugs. In the present study, steady state and time-resolved fluorescence measurements provide important structural and dynamical insights into the free and bound states of the therapeutically potent plant flavonoid fisetin (3,3′,4′,7-tetrahydroxyflavone) in a G_4_ DNA matrix. The excited state intra-molecular proton transfer (ESPT) of fisetin plays an important role in observing and understanding the binding of fisetin with the G_4_ DNA. Differential absorption spectra, thermal melting, and circular dichroism spectroscopic studies provide evidences for the formation of G_4_ DNA and size exclusion chromatography (SEC) proves the binding and 1∶1 stoichiometry of fisetin in the DNA matrix. Comparative analysis of binding in the presence of EtBr proves that fisetin favors binding at the face of the G-quartet, mostly along the diagonal loop. Time resolved fluorescence anisotropy decay analysis indicates the increase in the restrictions in motion from the free to bound fisetin. We have also investigated the fingerprints of the binding of fisetin in the antiparallel quadruplex using Raman spectroscopy. Preliminary results indicate fisetin to be a prospective candidate as a G_4_ ligand.

## Introduction

**Figure 1:**
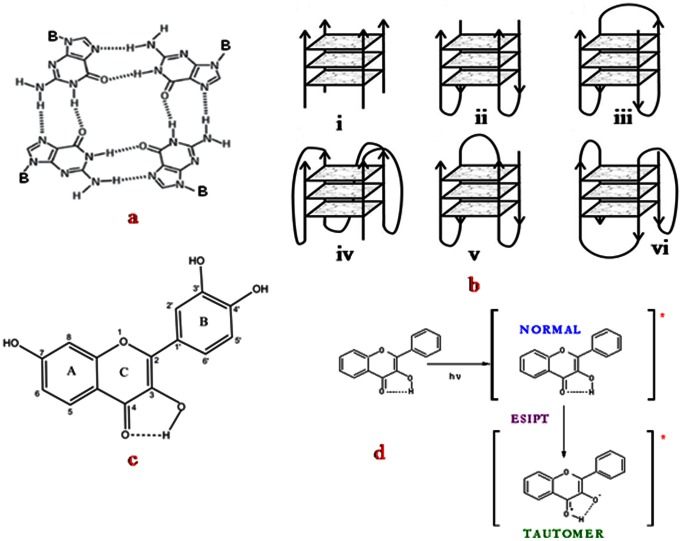
Structures of (a) G-quartet, four guanines can hydrogen bond in a square arrangement to form a G-quartet. There are two hydrogen bonds on each side of the square; (b) i. Tetramolecular parallel, ii. Bimolecular antiparallel structure with parallel adjacent strands, iii. Unimolecular antiparallel with alternating parallel strands, iv. Unimolecular parallel structure with three double chain reversal loops, v. Unimolecular antiparallel structure with parallel adjacent strands and a diagonal loop, vi. Unimolecular mixed structure with three parallel and one antiparallel strands. Structures b_iv-vi_ have been observed for the human telomeric repeat [4]; (c) Fisetin; (d) Ground and excited (denoted by *) states of normal (N) and tautomer (T) forms of a flavonol.

A little over a hundred years ago, in 1910, the German scientist I. Bang [1] first identified the unusual behavior of the nucleic acid base guanine (G) where he observed that only millimolar concentrations of guanine formed a gel in aqueous solution. But the reason for the gel formation as well as the structure of the gel were not known until 1962, when M. Gellert et al. [2] found that guanine bases can form a novel Hoogstein hydrogen-bonded tetrameric structure known as G-quartet (G_4_, Figure 1) where a total of eight hydrogen bonds form between four bases with an average of two bonds per base. These tetrads π-stack on each other like base pairs in a double helix, creating three-dimensional structures known as G-quadruplexes (G_4_) [3], [4] with various quadruplex folds as shown in Figure 1.

G_4_ is regarded as an important drug design target for the treatment of various human disorders. G_4_ forming sequences are prevalent in human genome, which includes many important regions of the eukaryotic genome, such as telomere ends, regulatory regions of many oncogenes c-kit [4], [5], proto-oncogene c-myc [6], Kirsten rat sarcoma viral oncogene homolog (KRas) [7], and vascular endothelial growth factors (VEGF) [8], suggesting their important role *in vivo*. The c-*myc* proto-oncogene is a key component of normal cell growth and differentiation. Telomeric DNA ends as the single strand (GGGTTA)_n_ overhang in vertebrates [9]. Telomerase is the enzyme responsible for the elongation of telomeres in tumor cells. The activity of telomerase is 85% more in immortal cancer cells, compared to normal cells, suggesting a relationship between telomerase activity and cancer cells [4], [9]. Literature data show that G_4_ ligands can effectively inhibit both the catalytic and capping functions of telomerase through stabilization of G_4_ [9], thereby driving the malignant cells toward apoptosis. However, although G_4_ sequences are rich in the genome, human telomeric DNA and proto-oncogenes have the higher potential to form G_4_ DNA, compared to tumor-suppressor genes [10], suggesting the possibility of treating cancer cells distinctively with effective G_4_-ligands. This opens the door to small-molecule based ligand- telomeric G_4_ in cancer therapy. There are evidences of efforts to find effective G_4_-ligands from natural products [11]–[13]. However, to our knowledge, till date, there are no reports describing systematic investigations on the role of plant flavonoids as G_4_ ligands. The flavonol fisetin (3,3′,4′,7-tetrahydroxyflavone, Figure 1) is a dietary flavonoid, present in a number of commonly eaten foods, such as strawberries, vegetables, nuts and wine, [14]–[16] and has been reported to protect nerve cells from oxidative stress-induced death and promote the differentiation of nerve cells [17]. *In vivo*, fisetin has recently been shown to possess interesting anticancer activity including lung carcinoma [18], and prostate tumours [19]. The high potency and low systemic toxicity of fisetin *in vivo* on one hand, and its exquisitely sensitive two color intrinsic fluorescence properties on the other hand, motivated us to undertake a detailed investigation on the binding and photophysical properties of fisetin in a G-rich single stranded DNA oligonucleotide with the 28 bases long sequence d(T_2_AG_4_)_4_ (in the 5′→ 3′)_._ We have used steady state and time resolved optical spectroscopic and chromatographic techniques. The formation of quadruplex is evidenced from the thermal difference absorption, UV melting and circular dichroism (CDs) spectra. It is also of interest to find out how the rotational dynamics of the ligand changes when it binds with its target. Hence time resolved fluorescence anisotropy decay studies were performed for fisetin in DNA matrix,and also in fluid methanolic solution taken as a reference. The rotational time constant (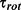) is determined by the size, shape, and flexibility of the ligand as well as the macromolecule. Size exclusion chromatographic studies provide evidence of the folded nature of the d(T_2_AG_4_)_4_ establishing the intramolecular nature of the quadruplex as well as the stoichiometry of the binding of fisetin in it. Raman spectroscopy provide useful insights regarding the antiparallel nature of the quadruplex DNA as well as the stability in the presence of fisetin. The purpose of this study is to examine the influence and stability of a medicinally potent drug fisetin in a quadruplex matrix in order to get a better insight into the factors regulating the formation and stabilization of the G_4_ structure. The information thus obtained is likely to be useful for the development of new G-quadruplex-based therapeutic agents.

## Materials and Methods

Fisetin and d(T_2_AG_4_)_4_ oligonucleotide were purchased from Aldrich Chemical Company and Integrated DNA Technologies respectively and were used as obtained. The solvents used were of spectroscopic grade and checked for any absorbing and/or fluorescent impurities. The final concentration of fisetin was kept in the order of 10^−6 ^M and methanol concentration was below 1% (v/v). The desalted oligonucleotide d(T_2_AG_4_)_4_ was dissolved in 10 mM Tris-HCl, pH 7.4 buffer, containing 25 mM NaCl made with deionized water. DNA concentration was determined by UV absorption measurement using molar extinction coefficient based on the nearest-neighbor approximation. 10 mM Tris-HCl, pH 7.4 buffer, containing 25 mM NaCl was used for all experiments with DNA except for size exclusion chromatographic (SEC) measurement, where 10 mM Tris-HCl buffer containing 100 mM NaCl was used. We chose the [NaCl] ∼ 25 mM following the range of salt concentration used by Vaughn et al. [6]. To prepare each solution for spectroscopic measurements, requisite amounts of flavonol from concentrated methanolic stock solution were added to the DNA solution and mixed with gentle shaking for a few minutes. Samples prepared for spectroscopic measurements were then equilibrated at room temperature (25°C) for 1 hour in dark prior to spectroscopic measurements.

### UV/Vis Absorption and Fluorescence Spectroscopy

Steady state absorption spectra were recorded with a Shimadzu UV 2550 spectrophotometer with Peltier temperature controller and 8-micro cell holder accessories used for melting studies, with 1°C/min and a wait period of 240 secs. Steady state fluorescence measurements were carried out with Shimadzu RF5301 (equipped with a Fisher temperature controlled accessory) spectrofluorometer.

Time resolved fluorescence decay measurements were performed using a Jobin–Yvon nanosecond time correlated single photon counting (TCSPC) setup. As excitation source a 375 nm laser diode having pulse FWHM ∼ 170 ps was used. An emission monochromator was used to block scattered light and isolate the emission. Data analyses were performed using DAS6 Fluorescence Decay Analysis Software, provided with the TCSPC instrument and were fitted with a multi exponential decay function,
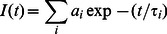
(1)where *a*_i_ and τ*_i_* represent the amplitudes and decay times respectively of the individual components for multi-exponential decay profiles. The goodness of fit was estimated by using reduced *χ^2^ (*namely 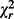 ) values as well as Durbin–Watson parameters (DW). A fit is considered acceptable for a given set of observed data and chosen function, when the 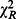 value is in the range 0.8–1.2 and the DW value is greater than 1.7, 1.75 and 1.8 for a single, double and triple exponential fit respectively [20], [21]. Average lifetime (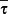) was calculated using the equation,
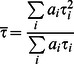
(2)where *a*_i_ and τ*_i_* represent the amplitude and decay time respectively of the individual components for multi-exponential decay profiles.

### Fluorescence Anisotropy

For measurement of fluorescence anisotropy, depolarization decays parallel (*I*_VV_) and perpendicular (*I*_VH_) were collected in an alternating manner for equal amounts of time until at least 10,000 fluorescence counts were collected in the peak channel of *I*_VV_. The time -dependent fluorescence anisotropy, *r*(*t*) was then calculated from the above data using the following relation [20], [21],
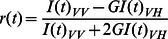
(3)where the G factor is defined as.
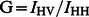
(4)and the intensities I_HV_ and I_HH_ now refer to the vertical and horizontal positions of the emission polarizer, with the excitation polarizer being horizontal. The value of G has been used as 0.56 as identified for the instrument [20], [21]. The fluorescence lifetime was determined by recording the decay at magic angle 54.7° polarization [20], [21]. In the case of spherically symmetrical molecules, free to rotate and excited with polarized light, anisotropy will decay with time following the equation
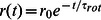
(5)where r_0_ is the limiting anisotropy, which is the anisotropy measured at the instant of photo selection at t = 0 and τrot is the rotational correlation time, which is an indicator of how rapidly a molecule rotates [21]. If molecular rotation is occurring, we expect that the anisotropy value will decay from an initial value (r_0_) to a randomized state (r_∞_) due to depolarization of the system. The same software was used for time resolved fluorescence intensity as well as fluorescence anisotropy decay analyses. Three independent measurements were performed on the fluorescence decay experiments and the averaged values are given in Tables 1 and 2.

**Table 1:** Fluorescence decay parameters of fisetin tautomer (Fis (T))^a^ and fisetin normal (Fis (N))^b^ species in water and in the presence of QD DNA.

Sample	*τ_1_*(ns)	*τ_2_*(ns)	*τ_3_*(ns)	*α_1_*	*α_2_*	*α_3_*	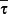 (ns)	*χ^2^_r_*	DW
Fis (T)^a^ in water	0.044	1.11	–	0.937	0.063	–	0.715	1.19	1.76
Fis (T)^a^ +7.5 µM QD-DNA	1.06	3.94	7.17	0.641	0.201	0.158	4.59	1.2	1.82
Fis (T)^a^ +27 µMQD-DNA	0.92	3.21	6.84	0.369	0.318	0.313	5.21	1.16	1.81
Fis (N)^b^ in water	0.011	0.733	–	0.998	0.002	–	0.096	1.16	1.85
Fis (N)^b^ +7.5 µM QD-DNA	0.17	0.69	3.51	0.61	0.38	0.01	0.80	1.14	1.71
Fis (N)^b^ +27 µMQD-DNA	0.2	0.71	3.82	0.724	0.261	0.015	0.98	1.2	1.58

a (λ_ex_ –375 nm_,_ λ_em_ - 550 nm), ^b^ (λ_ex_ - 375 nm_,_ λ_em_ - 450 nm),
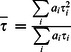
_._

[Fisetin] = 7.5 µM, QD-DNA solutions were prepared in 10 mM pH 7 TRIS buffer containing 25 mM NaCl. Three independent measurements were performed on the fluorescence decay experiments and the averaged values are given here.

**Table 2:** Parameters of Rotational Dynamics of Fisetin tautomer (Fis (T))^a^ and normal (Fis (N))^b^ species in water and in the presence of QD DNA.

Sample	*τ_rot 1_*(ns)	*τ_rot2_* (ns)	*α_1_*	*α_2_*	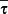 (ns)	*χ^2^_r_*	DW	*r_0_*	*r_∞_*
Fis (T) a in MeOH	0.205	–	1.0	–	0.205	0.99	1.79	0.28	0
Fis (T) a +27 µMQD DNA	16.79	–	1.0	–	16.79	1.10	1.79	0.29	0.09
Fis (N) ^b^ in MeOH	0.139	–	1.0	–	0.139	1.16	1.62	0.25	0.0
Fis (N) ^b^ +27 µM QD DNA	0.501	–	1.0	–	0.501	1.04	2.04	0.29	0.12

a (λ_ex_ –375 nm_,_ λ_em_ - 550 nm), ^b^ (λ_ex_ - 375 nm_,_ λ_em_ - 450 nm), FWHM ≈ 115 ps,
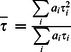
_._

QD DNA solutions were prepared in pH 7.0, 10 mM TRIS buffer containing 25 mM NaCl, [Fisetin] = 15 µM. Three independent measurements were performed on the fluorescence decay experiments and the averaged values are given here.

### Circular Dichroism Spectroscopy

Circular dichroism spectra were acquired with a Biologic Science Instruments (France) spectropolarimeter, using a rectangular cuvette with 1 cm path length. The scan rate was 60 nm/min, and five consecutive spectra were averaged to produce the final spectrum. All spectral measurements were performed at 25°C. The highest concentration of DNA for fluorescence decay, anisotropy (steady state, time resolved), and circular dichroism experiments were kept at ∼20 µM in order to avoid scattering and related artifacts.

### Raman Spectroscopy

Raman spectra of sample solutions were collected using quartz capillary tubes with a confocal Micro Raman spectrometer (model: LabRAM HR Vis. Horiba Jobin Yvon SAS France) at laser power 18 mWatt, with exposure 20 secs and repetitions 10, extending over a spectral scan range of 500–1800 cm^−1.^ The excitation wavelength used was 632.81 nm (using He Ne laser), grating 1800 grooves/mm, 100× objective having numerical aperture (NA) 0.9 giving the best confocality i.e. the depth of focus ∼2 µm, where there is no loss of Raman signal with the confocal hole size 200 µm.

### Size Exclusion Chromatography (SEC)

SEC used a 300 x 7.8 mm i.d. column (BioSep, 3000, Phenomenex) on an HPLC system (SCL 10A VP, Shimadzu) using a 10 mM tris buffer at pH = 7 with 100 mM NaCl to minimize matrix adsorption [22]. For the thymine oligonucleotides dT_5_, dT_10_, dT_15_, dT_20_, dT_30_, the averages of the retention times and the corresponding molecular masses were fitted linearly, from which the folded nature of the d(T_2_AG_4_)_4_ DNA was determined, assuming that there is no secondary interaction within the thymine oligonucleotides. It is pertinent to mention here that we have used the molar masses of the thymine oligonucleotides as standards for drawing a calibration plot, in order to obtain the molecular mass of the free and bound DNA for stiochiometric purpose. Absorption and fluorescence measurements of the species were made using the SPD-10AVi and RF-10AXL Shimadzu detectors, respectively. The time difference between the two detectors was determined from the absorption (260 nm) and the emission (*λ*_ex_ = 307 nm, *λ*_em_ = 370 nm) for the oligonucleotide 5′-CAGCA*GCAG-3′, where A* is 2-aminopurine. The injection volume was 20 *µ*L. Three or more chromatographs were acquired to determine an average retention time.

## Results and Discussion

### Circular Dichroism, UV Melting and Thermal Differential Absorption and Size Exclusion Chromatography

**Figure 2:**
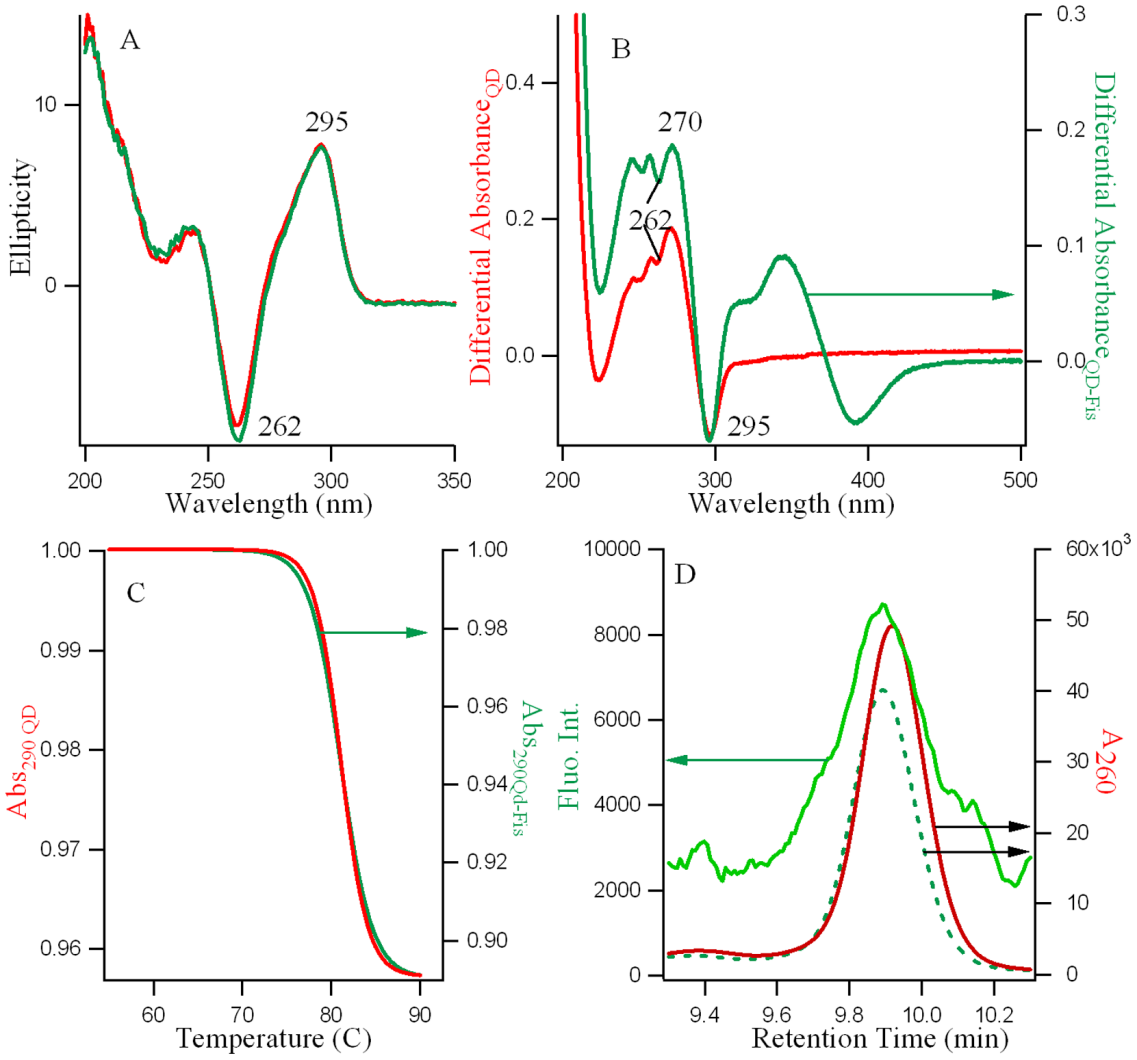
Red and green denote free and fisetin bound (T_2_AG_4_)_4_ DNA respectively in 10 mM Tris buffer, pH 7.4 with 10 mM NaCl. A: Circular Dichroism spectra of free DNA (20 µM, red) and in the presence of fisetin (20 µM, green), B: Thermal Difference Spectra of 5 µM of free DNA (red) and in the presence of 5 µM of fisetin (green), C: Typical UV melting profiles of the same as mentioned in B, monitored at 290 nm, where heating rate was 1°C min^−1^. D: Size-exclusion chromatograms of 5 µM solution of d(T_2_AG_4_)_4_ oligonucleotide, studied by absorbance at 260 nm, with (green dashed) and without (red solid) fisetin (5 µM). Using fluorescence emission the SEC chromatogram of the conjugate was also studied (green solid). The time difference of 0.06 mins between the fluorescence and absorption due to the time lag between the two detectors (see the Experimental section^21^) is corrected here. The experiments were performed in 10 mM Tris-HCl buffer, pH 7.4, 100 mM NaCl. Dilutions do not influence the retention times. Please refer to text and Figure S1 in File S1 for further details.

Circular dichroism (CD) spectra providea diagnostic signature for the structure of a G-quadruplex. The CD spectrum probes the asymmetry of bases where at wavelengths >200 nm, the asymmetry arises as a result of stacking interactions of bases [23], [24] and coupling with backbone transitions. Figure 2A shows the circular dichroic spectra of the d(T_2_AG_4_)_4_ with (green) and without (red) fisetin. It is a known fact that a peak around 260 nm and a trough around 240 nm implies the presence of a parallel G-quadruplex structure and a peak around 295 nm with a trough around 260 nm generally implies an antiparallel G-quadruplex [4], [23], [24]. Figure 2A depicts that in the absence of fisetin (red), the CD profile of the oligonucleotide d(T_2_AG_4_)_4_ shows a positive peak at 295 nm and a negative peak at 262 nm. These spectral positions and their relative ellipticities are consistent with base arrangements which form antiparallel quadruplex structures implying the formation of the same in the present case. It is pertinent to mention that in antiparallel structures, heteropolar head-head/tail-tail base stacking of G-quartets take place [24]. In the presence of fisetin (green profile), there is no significant change in the CD spectral features of the DNA, indicating that, the base stacking of the G-quartet is conserved, highlighting the non-invasive nature of fisetin binding in quadruplex matrix.

**Figure 3:**
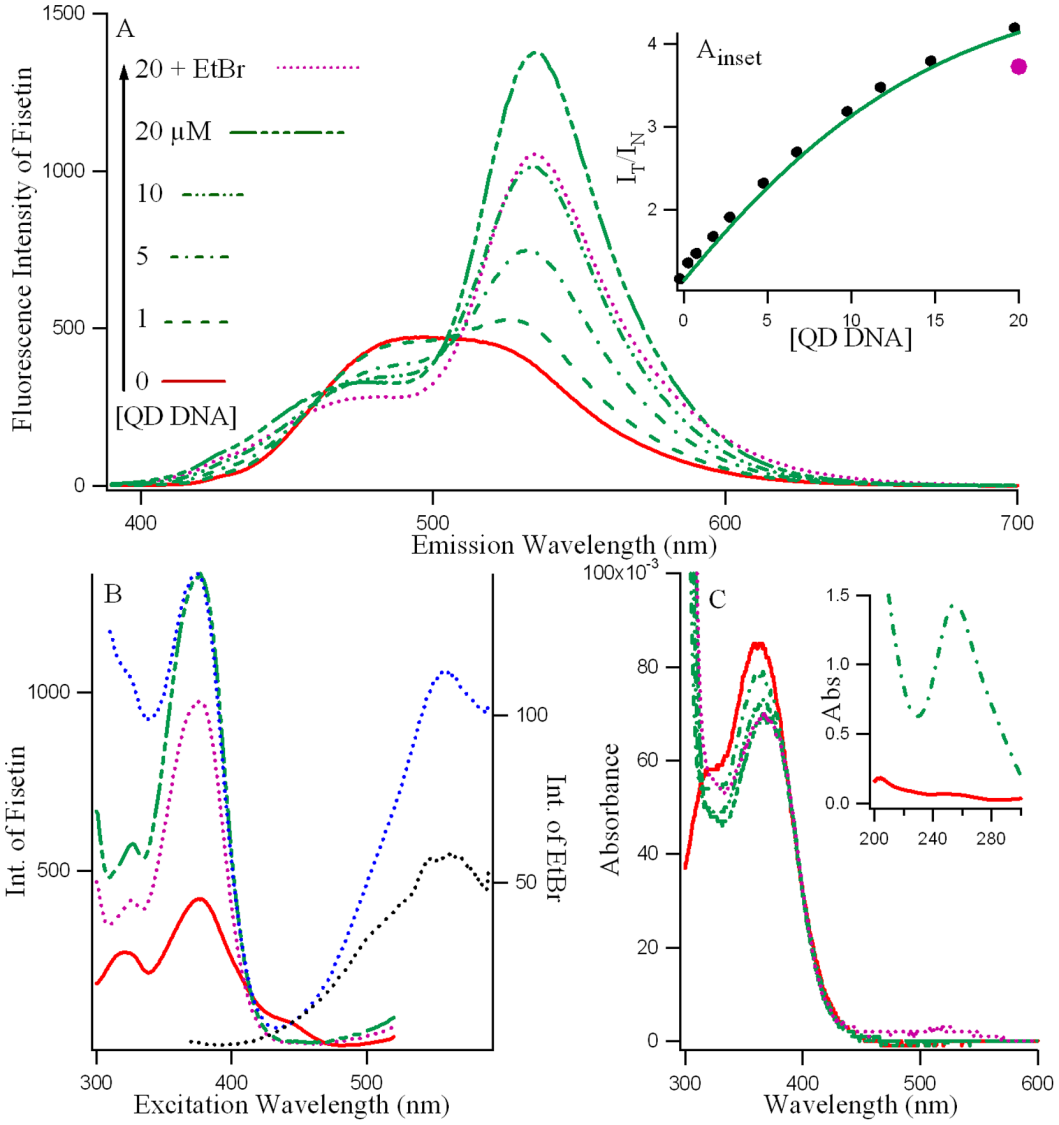
Red and green denote free and d(T_2_AG_4_)_4_ bound fisetin respectively in 10 mM Tris buffer, pH 7.4 with 25 mM NaCl. A: Fluorescence emission spectra (λ_ex_ = 370 nm); B: Fluorescence excitation spectra (λ_em_ = 530 nm); and C: Absorption spectra of fisetin (∼ 7.5 µM); in the presence of increasing concentrations of DNA (0― red, rest green; 1–, 5–·–, 10–··–, 20 µM–···–). A_inset_: Variation of the ratio of the intensity of tautomer vs normal isomers of fisetin (I_T at 530_/I_N at 470_) with increasing [DNA]. C_inset_ highlights the absorption spectra of fisetin bound 5 µM DNA (green) between 200–300 nm. Pink (…….) spectra and marker in Figures A, B, C and A-inset provide the fluorescence emission, excitation, absorption spectra and I_T at 530_/I_N at 470_ of 7.5 µM fisetin in 20 µM (T_2_AG_4_)_4_ in the presence of 7.5 µM of ethidium bromide (EtBr) respectively. The blue and black dotted lines in Figure 3B represent the excitation spectra of EtBr (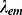 = 600 nm) with and without fisetin in 5 µM DNA.

The UV absorption of nucleic acids from 200 to 300 nm (e.g. Figure 3C inset) is exclusively due to transitions of the planar purine and pyrimidine bases [23]–[25]. They actually correspond to a number of different π → π* and n → π* transitions. The phosphate backbone contributes at ∼190 nm. Figure 2B and Figure 2C show the thermal difference spectra (TDS) [23], [25] and the corresponding thermal denaturation profiles at 290 nm of free and conjugate DNA respectively. The curves in Figure 2B are simply the result of the arithmetic difference between high (90°C) and low temperature (20°C) absorption spectra, obtained by subtracting the low temperature spectrum from the high temperature spectrum. These spectra provide information complementary to circular dichroism [23]. As is evident from Figure 2B, the spectral difference between the unfolded and the folded form which is the differential spectra in Tris buffer with NaCl (Figure 2B, red), shows two positive maxima at ∼ 244 and 270 nm, a shoulder at 257 nm and a negative minimum at 295 nm. Differential spectra have a typical and unique shape for quadruplexes [23], [25] and the characteristics of the spectral profile observed here are clearly supportive of the formation of quadruplex structure for d(T_2_AG_4_)_4_ DNA in 10 mM Tris buffer, pH 7.4 with 25 mM NaCl. In the presence of fisetin (the green profile in Figure 2B), the unique features of the TDS profile were protected, establishing the positive influence of the therapeutic agent in the quadruplex matrix. Furthermore, the occurrence of the induced positive peak at 345 nm with a shoulder at 312 nm along with the trough at 390 nm suggest the presence of fisetin inside the DNA matrix and the possible dipolar interactions between them.

Hypochromicity at 290 nm with increasing temperature in Figure 2C is mainly the result of base stacking [23]. The relative positioning of the bases play a crucial role in the absorption of light and the melting profile upon denaturation is attributed to the interaction between the dipoles induced in the chromophore by the light. Hypochromism at a given wavelength will not only depend on the intrinsic transition moments of each base, but also on the relative moment of the interacting bases (base-paired or stacked). The considerable hypochromic shift at 290 nm with increasing temperature in Figure 2C is indicative of quadruplex structures [4], [23] and the midpoint of the melting transition is the melting temperature, T_m_. As is shown in Figure 2C, there is no appreciable change in the T_m_ of the DNA structure in the presence of fisetin (both the Y-axes are normalized). The observed T_m_ of ∼81°C is in close agreement with Fox and Rachwal [26]. In this context it should be noted that at high temperatures, the chance of thermal degradation of fisetin is rare, as is evident from the TDS profile of the conjugated DNA in Figure 2B.

The formation of quadruplex form of d(T_2_AG_4_)_4 _in 10 mM Tris buffer, pH 7.4 with 25 mM NaCl was confirmed through CD, thermal melting and differential absorption spectra where the antiparallel nature of the quadruplex was also established by CD. However it was unclear if the quadruplex was uni or multi-molecular. To find out the nature of the quadruplex, size exclusion chromatography (SEC) was carried out. Support for the monomeric form of d(T_2_AG_4_)_4_ is obtained from molecular mass measurements (see Figure S1 in File S1). Thymine oligonucleotides were used to relate observed retention times to molecular mass, as these homooligonucleotides favor single-stranded and unfolded conformations. Size exclusion chromatography coupled with absorbance at 260 nm and fluorescence detection demonstrates that the retention time of the free d(T_2_AG_4_)_4_ is 9.92 min which is between the retention times of dT_20_ (9.84 min) and dT_15_(10.05 min, see Figure S1 top in File S1). With the intrinsic mass of the 28 bases long oligonucleotide (dT_2_AG_4_)_4_ of 8891.8 g/mol, the retention time in SEC should have been around dT_30_, which is not the case. The retention time of the fisetin bound oligonucleotide as studied through absorbance at 260 nm and fluorescence is 9.88 mins. This is indicative of the folded nature of (dT_2_AC_4_)_4_ in the solution, making it to behave as a smaller sized oligonucleotide (such as dT_20_ and dT_15_). The presence of fisetin in the DNA makes it heavier, giving its retention time lesser than the free state (Figure 2D green compared to red). Relative to single-stranded thymine oligonucleotides (Figure S1, top and bottom in File S1), the calculated molecular mass from the linear fit for free and fisetin bound (dT_2_AG_4_)_4_ is found to be 5213 g/mol and 5476 g/mol with a difference of 263 g/mol. It is noteworthy that the molar mass of fisetin is 286.24 g/mol which strongly indicates that the binding stoichiometry between fisetin and (dT_2_AG_4_)_4_ should be 1∶1. Thus while the optical spectroscopic measurements proved the antiparallel nature of the quadruplex along with the non-invasive binding of fisetin, the chromatographic studies provide the quantitative picture for the unimolecular quadruplex formation event, establishing the stoichiometry of ligand : DNA as 1∶1.

### Steady State Fluorescence Spectroscopy

Figures 3 A, B, C present the fluorescence emission, fluorescence excitation and UV absorption spectra of fisetin with increasing concentration of d(T_2_AG_4_)_4_ DNA. It is evident that addition of DNA induces drastic changes in the emission behavior of fisetin. In aqueous medium, the fluorescence spectrum of fisetin exhibits strong overlap between the normal and tautomer emission bands [27]. With the addition of the d(T_2_AG_4_)_4_, dual fluorescence behavior is observed. The emission spectra of fisetin consist of ‘two color’ fluorescence bands, namely a yellow-green emission band along with a high energy band in the blue-violet region. The blue-violet fluorescence is assigned to the S_1_ (π π*)→S_0_ normal (N, non-proton transferred) emission, whereas the large Stokes shifted yellow-green fluorescence is attributed to emission from a tautomer (T) species generated by an excited state intramolecular proton transfer (ESPT) process occurring along the internal H-bond (C(4) = O–HO–C(3)) of the molecule (Figure 1d) [26] The “blue-violet” and “yellow-green” fluorescence emissions occur from N* and T* species respectively (Figure 1d). The intensity ratio of tautomer : normal fluorescence (I_T/_I_N_) increases rapidly with increasing [(T_2_AG_4_)]_4_ (shown in Figure 3A inset) until ∼10 µM, beyond which the rate of increase decreases. These observations can be rationalized in terms of interference with the internal H-bond of fisetin which permits the ESPT process. It is noteworthy that the emission profiles of fisetin recorded in the DNA matrix resemble the situation in aprotic environment where ESPT emission behavior is prominent [28]. Moreover, this is consistent with the spectral characteristics of the excitation profile (monitored for the PT fluorescence) which reveals a weak but clearly perceptible vibrational shoulder (Figure 3B) [28] typical of a predominantly aprotic environment. The absorption spectrum of fisetin (Figure 3C) after binding with DNA shows some change in 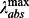 peak position (362 nm in buffer and 366 nm in 20 µM DNA) indicating the change in the microenviroenment of fisetin with no additional species being generated, which agrees well with our previous studies on fisetin in different environments [28]. The appreciable changes in absorption, excitation and emission spectra of fisetin from aqueous buffer to DNA matrix are indicative of the strong binding of fisetin with the G_4_ DNA. The enhanced tautomer emission as well as the strongly red shifted (∼ 15 nm for tautomer from 0 (520 nm) to 20 µM DNA (535 nm)) fluorescence band, indicate that the guest (fisetin) molecules experience relatively aprotic environments [28], in the DNA microenvironment (see Figure 3A). The normal emission (which possesses a strong charge transfer character and therefore susceptible to solvent dipolar relaxation induced spectral shifts) undergoes a blue shift of 14 nm with 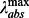
∼ 484 nm at 0 to ∼ 470 nm at 20 µM DNA suggesting the microenvironment inside DNA is hydrophobic too. This establishes fisetin as an excellent two color fluorescent probe to study its microenvironment. Time dependent emission study was performed on the conjugate of the fisetin and DNA over a period of four days and similar emission intensity suggested that the complex is stable for days (at least four days, data not shown).

In order to understand the mode of binding of fisetin in antiparallel intramolecular quadruplex matrix, we have exploited the well know extrinsic fluorescence probe ethidium bromide (EtBr), which mostly intercalates in DNA [29], [30]. Literature data provide evidence that EtBr binds with quadruplex mainly through intercalation or end stacking modes [29]–[31]. Figure 3A inset shows I_T_/I_N_ for fisetin in 20 µM DNA is the absence and presence of EtBr are 4.14 and 3.73 (pink dot) which suggest that in the presence of EtBr there is no major decrease in the fluorescence emission of fisetin, suggesting fisetin is still in the bound form inside the DNA matrix. The process of energy transfer is readily observed from the excitation spectrum of the energy acceptor [20], [32]. The blue and black dotted lines in Figure 3B show the fluorescence excitation of EtBr (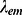 = 600 nm) in 20 µM DNA in the absence and presence of fisetin and the appearance of a band at 370 nm only in the latter case clearly indicate the existence of both fisetin and EtBr in DNA matrix and the occurrence of FRET from fisetin to EtBr which is intercalated between the adjacent guanine tetrads of the quadruplex. Figure S2 in File S1 presents the binding behaviors of EtBr and fisetin with the DNA in the presence of each other. As is shown in Figure S2 in File S1, the fluorescence of EtBr increases 4 fold upon binding with DNA and the addition of fisetin opens the path of energy transfer from fisetin to EtBr, thereby leading to a further increase (to a slight extent) in the emission intensity of EtBr (which now shows a 4.5 fold increase compared to EtBr alone). In the latter case, the appearance of the second band with 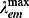 at ∼ 530 nm is attributed to the ESPT species of fisetin. Figures 3 and S2 suggest that the binding regions are adjacent for EtBr and fisetin in d(T_2_AG_4_)_4_ DNA matrix, which makes the FRET from fisetin to EtBr possible.

The possible binding site for fisetin is therefore the face of the G-quartet along the diagonal loop [33], [34], (see Figure 1 and text in the later sections) where possible π-delocalized system stacking is possible [33]. This agrees well the ΔAbs signal above 300 nm in Figure 2B (green profile). Furthermore, the absence of an induced CD signal above 300 nm in Figure 2A supports the possible binding of fisetin in the loop region rather than the groove region of the G_4_ matrix, because an induced CD signal >300 nm should have been observed in the later case [11].

### Time Resolved Fluorescence Studies: Fluorescence Decay Analysis by TCSPC Studies

Understanding the photophysical processes of a fluorescent probe in the excited state is made possible by time-resolved fluorescence decay studies. These were performed to examine how confinement in the DNA matrix influences the fluorescence decay profiles of the flavonoid fisetin when compared to the free state in an aqueous system. The normal species along with the ESPT tautomer emission decay kinetics of fisetin were studied in aqueous and d(T_2_AG_4_)_4_ DNA systems. The results are presented in Table 1. The fluorescence intensity decay of fisetin ESPT tautomer species in absence of DNA fits to a double exponential function with an average lifetime (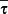) of 0.72 ns which is in agreement with a recent report [16]. Upon inclusion into DNA, there is a dramatic change in the decay kinetics of the fisetin tautomer species. In DNA, three discrete decay components are observed. Compared to the free state in aqueous system, 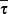 increases by six to seven times in 7.5 µM DNA (reaching a value of 4.59 ns) and 27 µM DNA (5.21 ns) environments. We observed 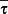 increases (∼ 14%) with increase in DNA concentration from 7.5 µM to 27 µM, along with an increase in the amplitudes of the corresponding individual decay components (Table 1). This indicates that the fisetin molecules experience relatively hydrophobic microenvironments within the DNA loop cavity where non-radiative decay processes are reduced [35], which is also reflected in the higher emission yield in Figure 3A. However, the changes in
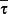
and population distribution of the normal species of fisetin are less significant in DNA matrix as displayed in Table 1. The nonexponential decay in the presence of high concentration of DNA indicates heterogeneity in the micro-environments of fisetin in the DNA matrix. It is pertinent to mention, in the diagonal loop region of the d(T_2_AG_4_)_4_ DNA, the thymine and adenine bases can form H-bond with the two –OH groups of B ring and one –OH group of A ring of fisetin (see Figure 1), thereby promoting the excited state intramolecular H-bond between C(3)-OH and C(4) = O and increasing tautomer population. The multiple decay components observed for the tautomer species of fisetin is likely to arise from populations differing in the extent of H-bonding within the microenvironment of the diagonal loop region of the intramolecular antiparallel quadruplex.

**Figure 4:**
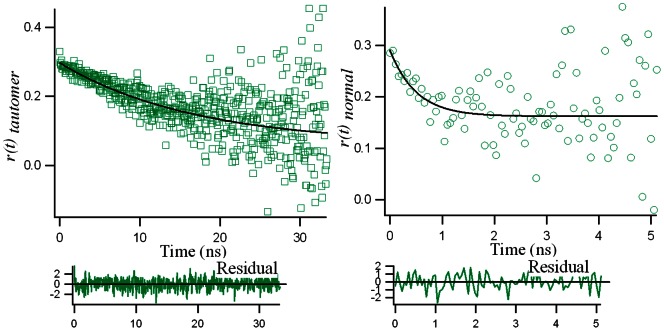
Fluorescence anisotropy decay *r(t)* for the fisetin tautomer (left) and normal species (right) in the d(T_2_AG_4_)_4_ DNA matrix at 25°C where the solid lines are the single exponential fit curves.

The fluorescence anisotropy decay parameters of fisetin (15 µM) in different media (in homogeneous solution of methanol (MeOH) and in DNA) are given in Table 2 and the decay profiles in DNA only (for both tautomer and normal) are shown in Figure 4. In MeOH and DNA matrix (27 µM), the anisotropy decay of fisetin tautomer is found to be single exponential with rotational time constant (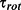) 0.21 ns and 16.79 ns, while for the normal species the rotational time constants are 0.14 ns and 0.5 ns (Table 2, Figure 4) respectively. Equation 5 states that if stochastic rotations occur in every axis of rotation with equal probability, the anisotropy will decay as a single-exponential function with a decay constant 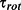 [36]. We observed the measured anisotropy decay to be fitted by a mono-exponential function which can be attributed to a spheroid molecular shape (because nonspherical fluorophores will rotate at different rates along different axes) of the fisetin bound quadruplex DNA [36]–[38]. 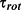 depends on the combined effect of three different kinds of motions, namely, (1) the wobbling motion of the fisetin molecule in the DNA (2) the lateral diffusion of the fisetin along a spherical surface; and (3) the rotational motion of the whole fisetin bound DNA [37], [38]. Compared to type 2 and 3 motions, the wobbling motion (Type 1) is much faster, occurring in the picoseconds range [36]–[38]. Here 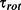
is attributed to the overall rotational motion of the whole fisetin bound DNA oligonucleotide in the solution. Considering the slow decay constant of the ESPT species (16.79 ns in Table 2) for fisetin in DNA, we can envision the overall conformation of fisetin in the bound state to be swelled compared to that in the free state. The increase in the rotational time constants from MeOH to DNA is due to the increase in the restrictions in the motion of the fisetin molecules in the DNA matrix [36] as well as the conformational swelling due to the overall motion of the conjugated DNA. Inside the DNA matrix, the fisetin molecule is locked through non-covalent interactions such as H-bonding, hydrophobic and van der Waals interactions, which make the rotation of fisetin difficult, thereby increasing the 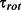 and constraining type 1 and 2 motions. The faster decay constants in MeOH solution for both normal and tautomer are due to the type 1 and 2 motions.

From the time resolved decay of the anisotropy (Figure 4), combined with equation (5) we obtained the value of the anisotropy at t = 0 (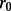), the final value (r_∞_) as well as 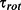. The anisotropy of fisetin emission decays from its initial r_0_ value (∼ 0.3 for both N, T) to a final value (r_∞_) of 0 in methanol (both N and T) and 0.12 (N) and 0.09 (T) in DNA (Table 2). It may be pertinent to mention here that the limiting anisotropy (r_0_) values measured experimentally by analyzing the time-resolved decay of anisotropy using equation (5) are often found to be lower than the fundamental anisotropy values predicted by theory (0.4 for single photon selection), which is attributed to the presence of depolarization factors [20], [36]. The depolarization process(es) responsible for the discrepancy between our measured r_0_ value and the limiting anisotropy (at t = 0) must occur on a timescale significantly faster than the time resolution of our instrument (in the order of 10^2^ ps) because they appear to take place instantaneously. The final value of anisotropy, *r_∞_* is 0 when molecular rotation is freely occurring, which is observed for fisetin N and T in methanol solution where the anisotropy value has decayed to 0, indicating that the orientation of the emission dipole of fisetin has randomized, as is expected. On the other hand, inside the DNA matrix, fisetin N and T forms have non-covalent interactions with the DNA matrix which hinders the rotational motion of the fluorophore, so that the orientation of the emission dipole does not randomize, and consequently the anisotropy value does not decay to zero [20], but instead assumes appreciable values (*r_∞_ = *0.09 for the tautomer,and 0.12 for the normal species). In this connection, it is noteworthy that in the ground state the N form of fisetin has more H-bonding interactions (involving the A, B, C rings’ –OH groups as well as C(4) = O where all H-bonds are internal) with the DNA matrix than the precursor of the ESPT-form (involving only the A, B rings’ –OH groups, where intramolecular H-bond between C(4) = O–HO–C(3) could occur leading to the ESPT), bringing the emission dipole of N more restricted than T form. This rationalizes the observation of the higher value for the limiting anisotropy *r_∞_* for the normal species.

### Raman Spectroscopy

**Figure 5:**
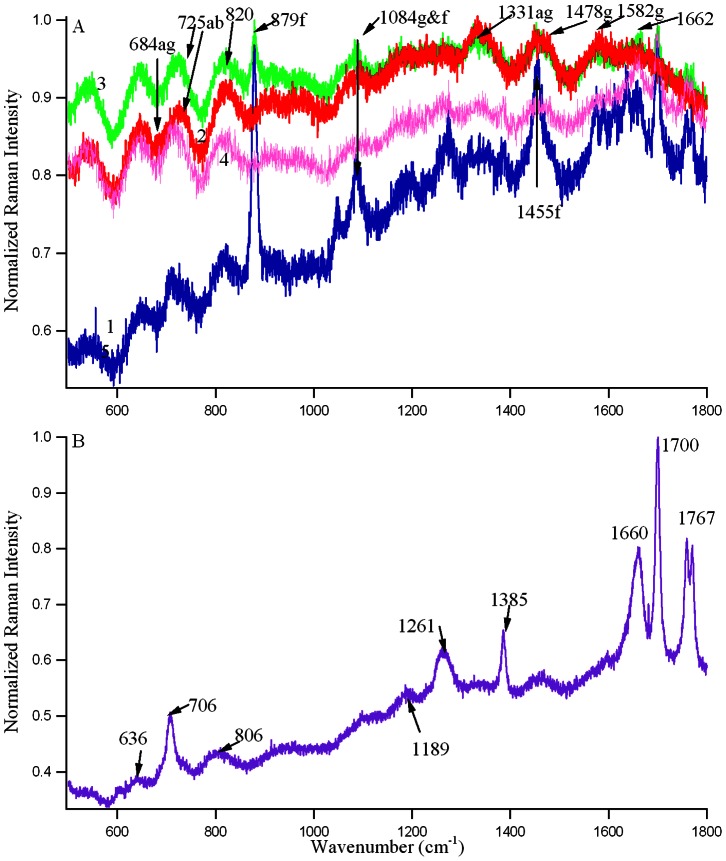
A: Typical Raman spectra of 1) blue-1 mM Fisetin, 2) red-1 mM (T_2_AG_4_)_4_ DNA, 3) green-1 mM (T_2_AG_4_)_4_ DNA conjugated with 1 mM fisetin, 4) pink-10 mM Tris buffer, pH 7 with 25 mM NaCl in which all the above solutions are prepared, B: 1 mM T_15_ DNA oligonucleotide dissolved in water. Here ab, tb, g, ag, f denote adenine base, thymine base, guanine tetrad, antiparallel guanine tetrad and fisetin respectively.

In order to understand the binding region of fisetin in the DNA, we recently initiated Raman spectroscopic studies and the results of the initial measurements are presented in Figure 5. To facilitate the comparison, the Raman spectra are normalized, simply by dividing the raw data by its maximum value, so that the highest positive peak has a Y-value of ∼+1. The Raman spectra of the quadruplex-forming d(T_2_AG_4_)_4_ were recorded both in the presence (green) and absence of fisetin (red) as shown in Figure 5A. The vibrational band around 1478 cm^−1^ is assigned to the C8 = N7–H2 bond deformation of the guanine tetrad d(T_2_AG4)_4_ and is therefore a marker for the presence of the quadruplex structure [39] in both the red and green profiles. It is noteworthy that the Raman peak around 1660 cm^−1^, corresponding to the C6 = O6 stretching mode of the DNA base (in this case guanine), is absent due to the presence of the H-bond between O6 and H1 in the quadruplex structure [39]. This C = O stretching vibrational band can be observed in the Raman spectra of thymine oligonucleotide (T_15_), in Figure 5B, where the DNA base is thymine, (purple profile in Figure 5, random single stranded conformation). The 1385 cm^−1^ band is also attributed to deoxythymidine, dT. In addition, the antiparallel topology is exhibited in the red and green profiles (free and conjugated DNA respectively) by the presence of the shoulders around 684 cm^−1^ and 1330 cm^−1^ (relative to the Tris buffer alone pink spectra), indicating C2′-*endo*/*anti* guanosine conformation [39]. The 1582 cm^−1^ band is also associated with the quadruplex form [39]. The 725 cm^−1^ band (assigned to the adenine ring breathing vibration) appears as only a shoulder in the red and green spectra since there is only one adenine residue in the quadruplex forming oligonucleotide sequence. The intense 1084 cm^−1^ band, which corresponds to the symmetric stretching of the ionized phosphate groups in the guanine nucleotides involved in the Hoogstein-base pairing, is also indicative of the presence of the guanine tetrad [39].

**Figure 6:**
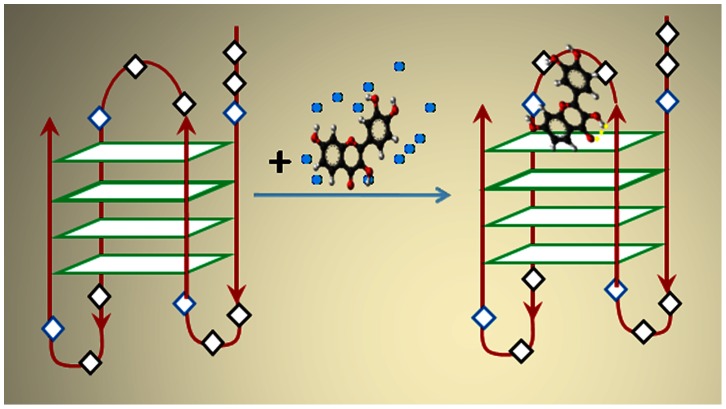
Left: Intramolecular antiparalel quadruplex with adjacent parallel strands and diagonal loop. T, A, G bases are denoted by black, blue and green blocks respectively. Right: Stacking interactions between the aromatic rings of fisetin and G-quartet in the loop region in a 1∶1 ratio. The blue dots represent water molecules surrounding fisetin in bulk buffer solution. The yellow dotted line represents the intramolecular H-bond in the fisetin leading to the formation of excited state tautomer inside the DNA matrix.

On the other hand, the Raman spectra of fisetin-DNA complex (green profile) presents two extra shoulders (not present in free DNA) at 879 cm^−1^ (HCCC bending of A, B rings [40]) and 1700 cm^−1^ (C = O stretching of C, and OC of B rings of fisetin [40]) which are the characteristic lines of fisetin alone (blue profile) showing the presence of fisetin in the DNA. The intensity of the 1084 cm^−1^ band which is a characteristic of G_4_ slightly increases in the conjugate DNA which also is evident of fisetin binding. The line at 1455 cm^−1^ for free fisetin (C-O, C-C stretching of B ring [40]) is totally absent in the conjugate profile, indicating the absence of free movement of B ring which can only be possible if the B ring interacts with the DNA matrix, via H-bond interaction. (Figure 5A). Since the major spectral changes between free and conjugated DNA occurred (except that for the presence of fisetin, discussed above) at 1662 (shift of thymine band from 1660 to 1662), 725, 820 cm^−1^, which are the adenine and thymine marker lines, [39]–[41] suggesting the binding of fisetin through the loop region which is made up of thymine and adenine bases, as is presented in Figure 6. This agrees well with the interpretations from CD, differential absorption and fluorescence spectroscopic results.

## Summary and Concluding Remarks

In summary, for the first time as far as we are aware, a systematic and thorough investigation, on the quadruplex forming DNA sequence and its binding with the therapeutically very potent plant flavonol fisetin, is provided using various spectroscopic and chromatographic measurements. The current study established that d(T_2_AG_4_)_4_ in a sodium ion-rich environment folds into an antiparallel intramolecular G-quadruplex consisting of four stacked G-quartets. The four strands are connected with two different types of loops: diagonal and edgewise consisting of 3 nucleotides. Thermal melting, differential absorption, CD, Raman spectroscopic along with size exclusion chromatographic measurements proved to be very useful in determining the structure of the quadruplex and the binding of fisetin in it. The structure and dynamics of the fisetin in the conjugated state is well understood exploiting steady state and time resolved fluorescence and anisotropic studies. We can envision that the interesting findings presented herein would provoke further extension of this research to other flavonoids of high potency and low systemic toxicity as ligands for G_4_ DNAs to treat various disorders of physiological system.

## Supporting Information

File S1 Figures S1 and S2 along with their legends. Figure S1 shows the comparative SEC chromatogram and linear fit of the retention times of the thymine size standards and quadruplex DNA. Figure S2 presents the fluorescence emission spectrum of EtBr with increasing [quadruplex DNA] in the absence and presence of fisetin.
(DOC)
