# Supplementary material for: Prospect of Bioflavonoid Fisetin as a Quadruplex DNA Ligand: A Biophysical Approach
Source: PLoS One. 2013 Jun 13;8(6):e65383. doi: 10.1371/journal.pone.0065383 (PMC3681855; doi:10.1371/journal.pone.0065383)
Supplement: File S1 — Figures S1 and S2 along with their legends. Figure S1 shows the comparative SEC chromatogram and linear fit of the retention times of the thymine size standards and quadruplex DNA. Figure S2 presents the fluorescence emission spectrum of EtBr with increasing [quadruplex DNA] in the absence and presence of fisetin. (DOC) [file pone.0065383.s001.doc]

**Prospect of bioflavonoid Fisetin as a Quadruplex DNA ligand: A Biophysical approach**

*Bidisha Sengupta^1*^, Biswapathik Pahari^2^, Laura Blackmon^1$^,*

*Pradeep K. Sengupta^2*^*

*^1^Department of Chemistry, Tougaloo College, 500 West County Line Road, MS, 39174, USA*

*^2^Biophysics Division, Saha Institute of Nuclear Physics, 1/AF Bidhannagar, Kolkata, 700064, West Bengal, India*

^*^ Corresponding authors:

Bidisha Sengupta, email: [bsengupta@tougaloo.edu](mailto:bsengupta@tougaloo.edu); bsgupta.tougaloo@gmail.com (phone: +1-601-977-7779, FAX: +1-601-977-7898)

Pradeep K. Sengupta, email: [pradeepsinp@yahoo.co.in](mailto:pradeepsinp@yahoo.co.in); [pradeepk.sengupta@saha.ac.in](mailto:pradeepk.sengupta@saha.ac.in) (phone:+919831393962)

^$^ Present address: Department of Microbiology, University of Mississippi, Jackson, MS


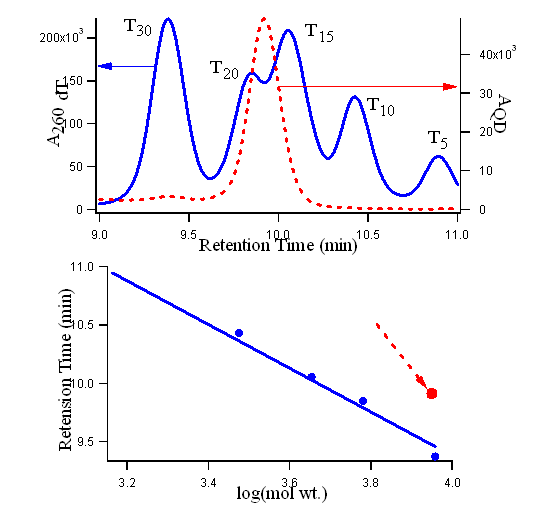


Figure S1. Top: The blue solid and red dashed lines denote the size exclusion chromatogram derived from absorbance at 260 nm of a mixture of single-stranded thymine oligonucleotides dT5, dT_10_, dT_15_, dT_20_, and dT_30_ (as size standards) and (dT_2_AG_4_)_4_ respectively. Bottom: The calibration curve of the size standards to relate the retention time on the column to the molecular mass. The mobile phase is 10 mM Tris buffer at pH 7.4 with 100 mM NaCl. The retention time of (dT_2_AG_4_)_4_ with the intrinsic molar mass is marked with the red solid circle, which is out of the linear fit in the calibration plot, displaying and indicating the folded nature of the G rich oligonucleotide.


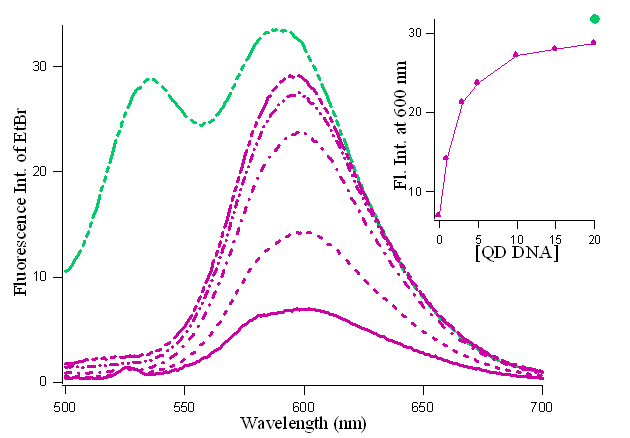


Figure S2. Fluorescence emission spectra of EtBr (pink, 7.5 µM, λ_ex_ = 480 nm) in buffer (solid), (T_2_AG_4_)_4_ DNA (5 ---, 10 -.-, 15 -..-, 20 µM -…-). Inset shows the plot of fluorescence intensity of EtBr at 600 nm with increasing [DNA]. Green -.-.- represent the emission spectrum of EtBr in 20 µM DNA in presence of 7.5 µM fisetin, the intensity of which is shown in green dot in inset.
